# Gradually progressive cholangiolocellular carcinoma: a case report

**DOI:** 10.1186/s40792-021-01342-0

**Published:** 2021-12-20

**Authors:** Kosuke Akiyama, Tomoyuki Abe, Akihiko Oshita, Akinori Shimizu, Keiji Hanada, Shuji Yonehara, Tsuyoshi Kobayashi, Hideki Ohdan, Toshio Noriyuki, Masahiro Nakahara

**Affiliations:** 1 Department of Surgery, Onomichi General Hospital, 1-10-23, Hirahara, Onomichi, Hiroshima 722-8508 Japan; 2 Department of Gastroenterology, Onomichi General Hospital, Onomichi, Hiroshima Japan; 3 Department of Pathology, Onomichi General Hospital, Onomichi, Hiroshima Japan; 4 Department of Gastroenterological and Transplant Surgery, Graduate School of Biomedical and Health Sciences, Hiroshima University, Hiroshima, Hiroshima Japan

**Keywords:** Cholangiolocellular carcinoma, Hemangioma, Liver resection

## Abstract

**Background:**

Cholangiolocellular carcinoma (CoCC) is a relatively rare primary liver tumor. We present a literature review and case report of a patient who presented with a slow-growing CoCC that was completely resected after a 5-year follow-up period.

**Case presentation:**

The patient was a 66-year-old man with a history of inflammatory thoracic and intra-abdominal pseudo-tumors. He was regularly followed up at our hospital for partial dilation of the pancreatic duct branch located in the body of the pancreas. Five years earlier, computed tomography (CT) demonstrated a small tumor in liver segment 4. Radiological findings were suggestive of hemangioma. Tumor size gradually increased during the 5-year follow-up period. CT scans showed that the tumor had progressed in size from 10 to 20 mm. Positron emission tomography CT revealed an accumulation of fluorodeoxyglucose (standardized uptake value max 5.3) at the tumor site. The tumor exhibited high intensity on T2-weighted and diffusion-weighted images of ethoxybenzyl magnetic resonance imaging. The tumor showed high intensity during the early phase but low intensity during the hepatobiliary phase. Tumor markers were within their respective normal ranges. Suspecting intrahepatic cholangiocarcinoma, left hepatectomy was performed. The tumor was diagnosed as CoCC based on pathological findings. The patient’s post-operative course was uneventful. The patient survived for a year, without any recurrence.

**Conclusions:**

In cases dealing with small tumor sizes, it is difficult to distinguish between CoCC and hemangioma due to their similar radiological findings. Thus, it is important to consider the diagnosis of CoCC in small benign hepatic tumors. As such, follow-up radiological examination is recommended.

## Background

Cholangiolocellular carcinoma (CoCC) is a rare tumor that accounts for approximately 1% of all primary liver tumors [1]. Steiner and Higginson et al. reported the first few cases of CoCC detected in 1959 [2]. They speculated that it originated from the canals of Hering or cholangioles occupied by hepatic progenitor cells (HPCs). The characteristic radiological findings of CoCC are very similar to those of intrahepatic cholangiocarcinoma (ICC) or hepatocellular carcinoma (HCC) [3, 4], making pre-operative diagnosis of CoCC clinically challenging. However, Arizumi et al. reported that the prognosis of CoCC after curative surgery was better than that of ICC [5]. Here, we report a literature review and a case of slow-growing CoCC derived from a normal liver.

## Case presentation

**Fig. 1:**
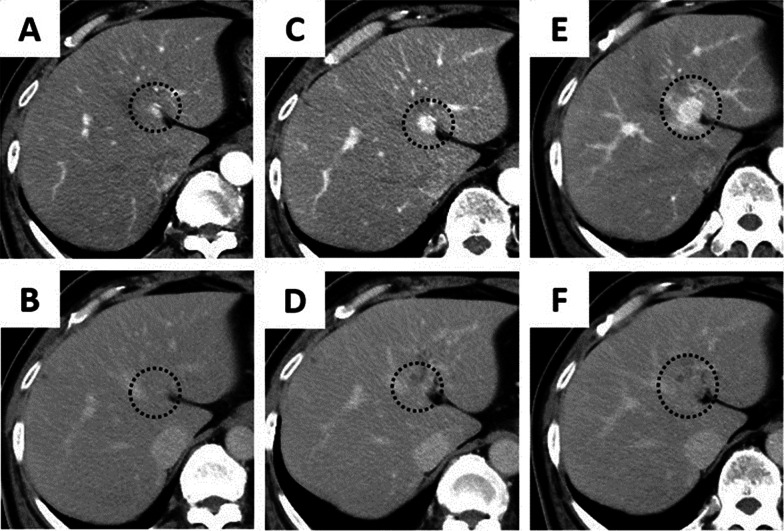
Dynamic abdominal computed tomography (CT) findings. **A** In 2015, the arterial phase showed enhancement of tumor at liver segment 4, measuring 10 mm in diameter. **B** The tumor was isodense during the late phase. **C** In 2017, the arterial phase showed enhancement of the tumor, measuring 15 mm in diameter. **D** The tumor remained isodense during the late phase. **E** In 2020, the arterial phase showed tumor progression, measuring 20 mm in diameter. The tumor was located close to the left first branch of Glisson. **F** The tumor remained isodense during the late phase

**Fig. 2:**
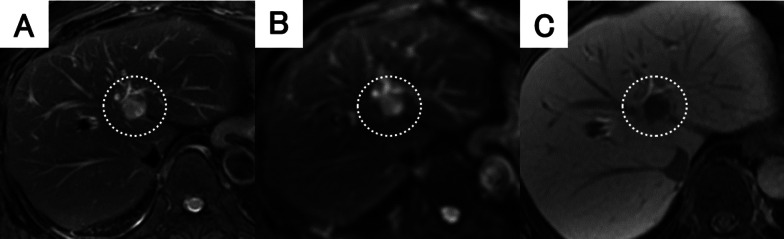
Findings of ethoxybenzyl magnetic resonance imaging (MRI). **A** The tumor had high signal intensity upon T2-weighted imaging. **B** Diffusion-weighted imaging showed high signal intensity of the tumor. **C** The tumor showed high intensity during the early phase and loss of primovist® uptake during the hepatocyte phase

**Fig. 3:**
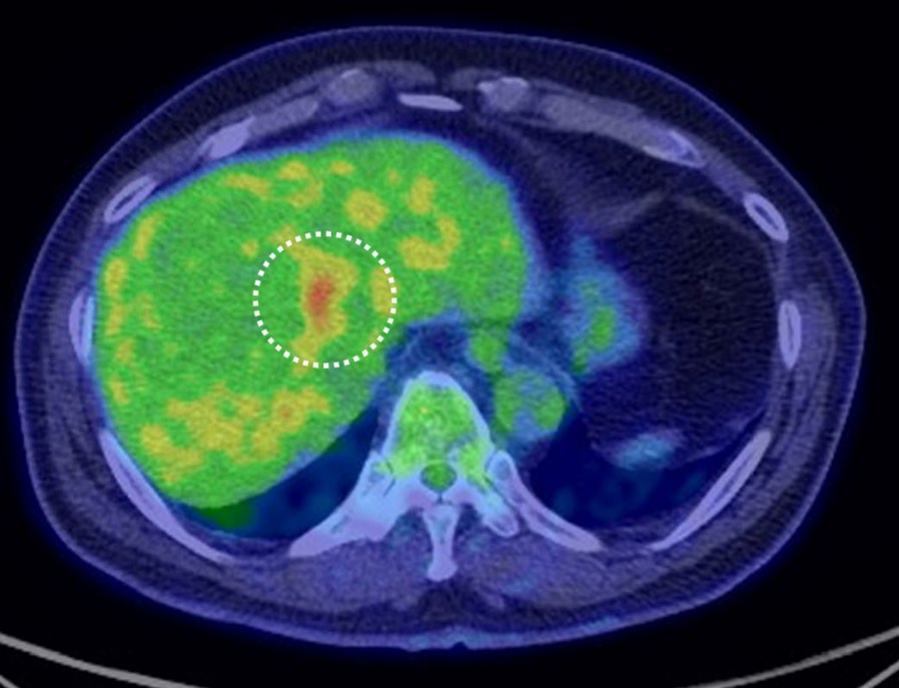
Positron emission tomography-computed tomography (CT) shows an accumulation of fluorodeoxyglucose (standardized uptake value max 5.3)

**Fig. 4:**
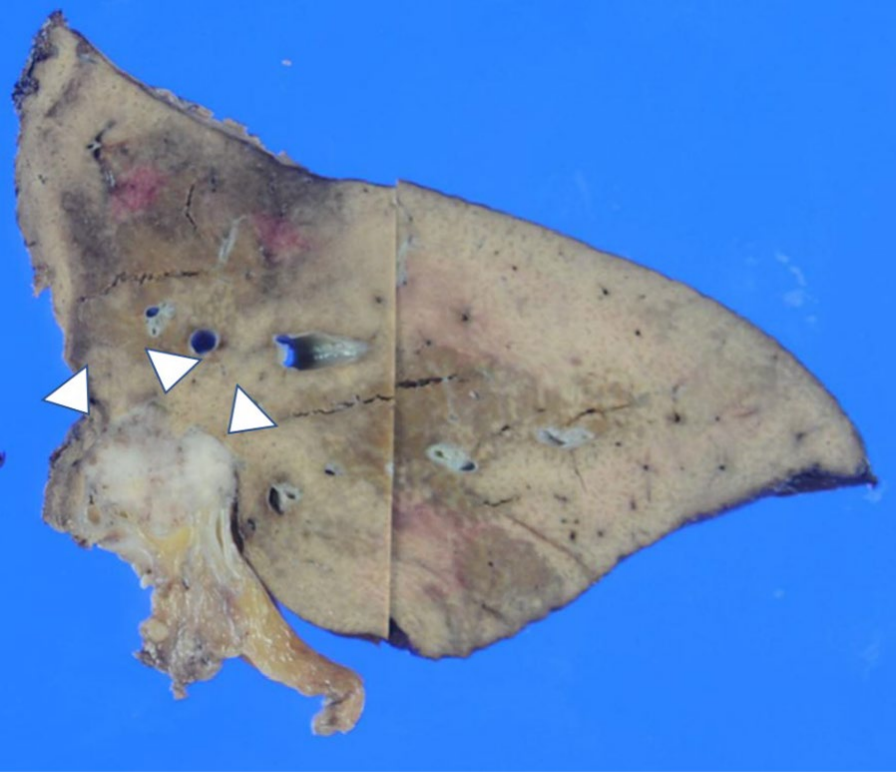
Macroscopic examination shows that the tumor was well-defined, grayish-white, and solid, measuring 15 × 15 mm in diameter

**Fig. 5:**
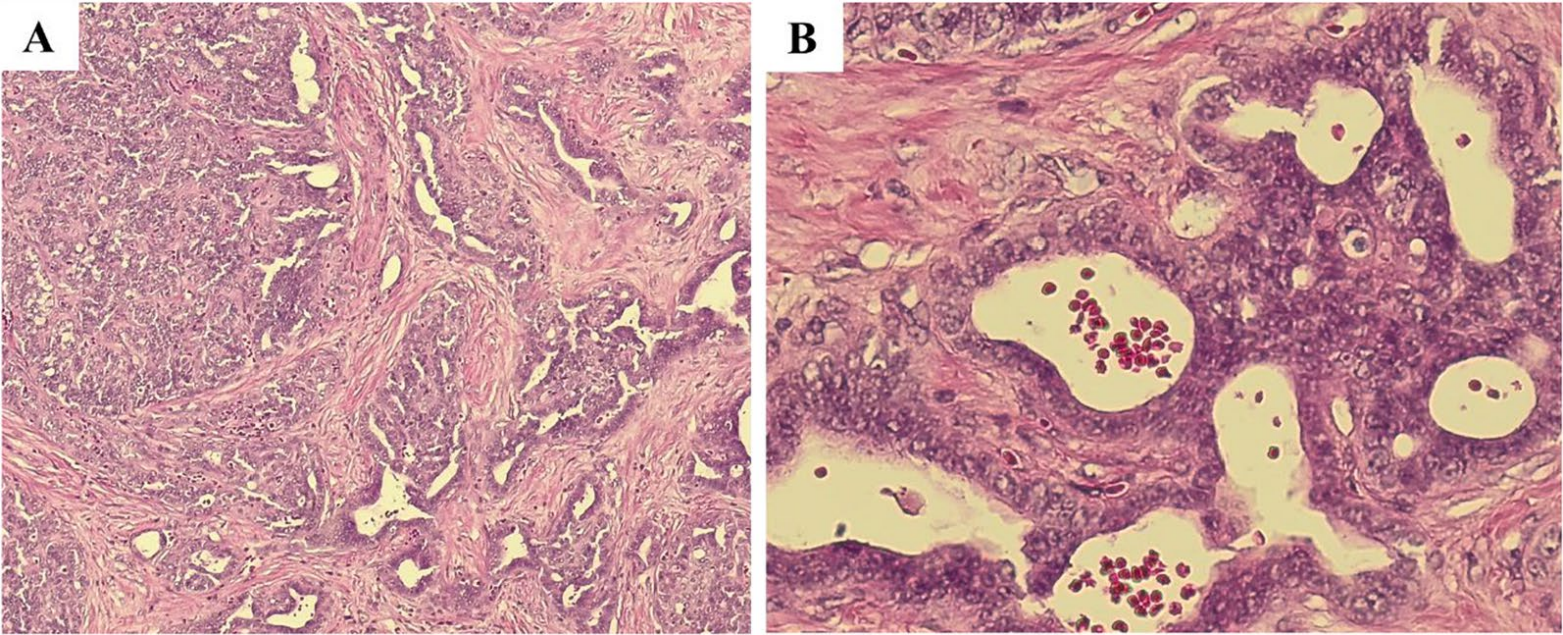
Microscopic findings with hematoxylin and eosin staining. **A**, **B** Atypical, poorly formed, cuboidal epithelium at the margins formed by large and small dilated tubular glands, suggesting a cholangioma-like morphology (**A** ×40, **B** ×200)

**Fig. 6:**
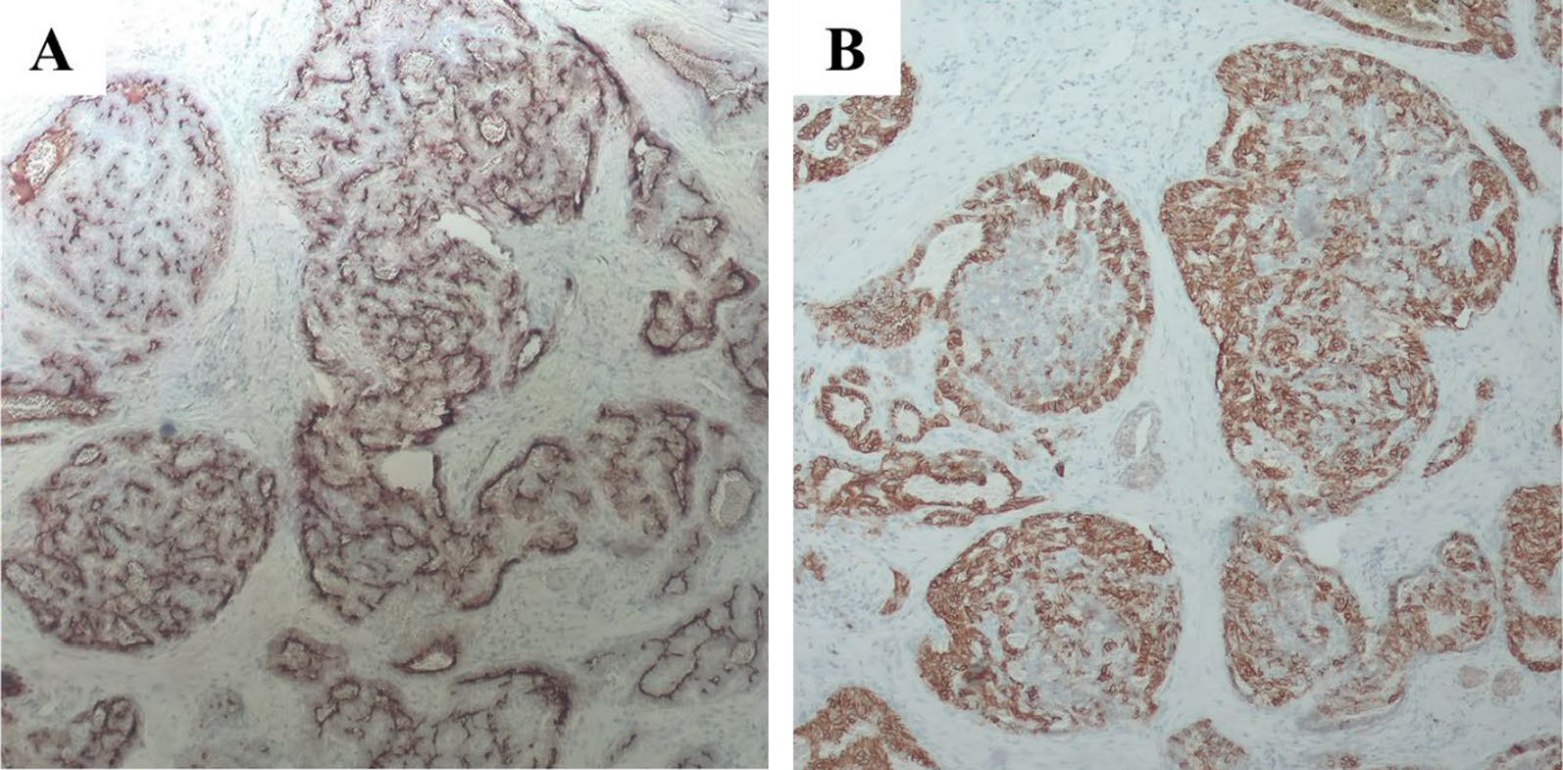
Findings of immunohistochemical staining. **A** Epithelial membrane antigen (EMA) was strongly stained in the membrane of the cancer duct, showing a characteristic membranous pattern. **B** Neural cell adhesion molecule 1 (NCAM1) was positive in the cytoplasm of the tumor cells (**A** ×40, **B** ×40)

The patient was a 66-year-old man with a history of cholelithiasis and inflammatory pseudo-tumors of the abdomen and chest wall. The patient was followed up every 6 months with MRCP and CT for the pancreatic branch type of intraductal papillary mucinous neoplasm. In 2015, computed tomography (CT) demonstrated a ~ 10 mm tumor in liver segment 4 with enhancement seen in the arterial phase (Fig. 1A, B). Based on the radiological findings, the tumor was highly suggestive of a hemangioma. As for additional testing, hepatitis B and C markers were negative, Child–Pugh grade was A, and liver damage was grade A. In addition, levels of tumor markers, such as carcinoembryonic antigen (CEA), carbohydrate antigen 19–9, and α-fetoprotein were within their respective normal ranges. The tumor gradually increased in size during the 5-year follow-up period (Fig. 1). CT revealed that the tumor size had progressed to 20 mm by 2020. The tumor showed enhancement during the arterial phase and iso-density during the late phase, which was located close to the left first branch of the Glisson (Fig. 1E, F). There was no lymph node metastasis seen around the hepatoduodenal ligament nor any distant metastases. On ethoxybenzyl magnetic resonance imaging (MRI), the tumor showed low signal intensity upon T1 weighted imaging, but high signal intensity upon T2 weighted and diffusion-weighted imaging (Fig. 2A, B). The tumor showed high intensity during the early phase and loss of primovist® uptake during the hepatocyte phase (Fig. 2C). No infiltration into surrounding vessels was observed. Positron emission tomography–CT identified an accumulation of fluorodeoxyglucose (standardized uptake value max 5.3) at the tumor site (Fig. 3). Based on these findings, the pre-operative diagnoses were HCC, ICC, and CoCC. Thereafter, left hepatectomy was performed. Intraoperatively, the liver was normal, and peritoneal seeding or ascites were not observed in the abdomen. The operative time was 424 min, and the intraoperative blood loss was 500 mL. On macroscopic examination, the tumor was well-defined, grayish-white, and solid. The tumor was 15 mm × 15 mm in diameter (Fig. 4). Microscopically, the atypical cuboidal epithelium became multi-nodular, forming small tubular glands and cord-like structures. Nuclear atypia was mild, and no mucus production was observed. Atypical, poorly formed cuboidal epithelium at the margins formed large and small dilated tubular glands, showing a cholangioma-like morphology (Fig. 5A, B). Immunohistochemical staining revealed that the cells tested positive for cytokeratin (CK)-7, CK-19, and CEA. Epithelial membrane antigen (EMA) was strongly stained in the membrane of the cancer duct, exhibiting a membranous pattern. The cytoplasm of tumor cells was positive for neural cell adhesion molecule 1 (NCAM1) (Fig. 6A, B). Based on these findings, the final pathology report revealed that the tumor was CoCC (T1N0M0, stage IA), according to the Japanese General Rules for the Clinical and Pathological Study of Primary Liver Cancer 6^th^ edition. On the 18th post-operative day, the patient was discharged without any complications. The post-operative course of the patient was uneventful, without any recurrence happening 1 year after the surgery.

## Conclusions

According to the World Health Organization (WHO) classification, CoCC is categorized as combined HCC and ICC with stem cell features and CoC subtype [6]. Recently, based on the 2019 WHO guidelines, CoCC has been classified as a subtype of small duct ICC [7]. The incidence of CoCC comprises 0.6% of all primary liver tumor cases [1, 2]. It has been reported that more than half of these cases are associated with viral hepatitis. Chronic inflammation and hepatic damage are strongly associated with CoCC occurrence [8]. Another theory on the origin of CoCC is the activation of HPCs due to hepatic damage and chronic stimulation [8].

A characteristic radiological finding of CoCC is a pattern of whole tumor enhancement during the early phase. Tumor enhancement has a slightly lower density in the late phase on dynamic CT. A pattern of ring enhancement at the tumor margins during the early phase with central enhancement during the late phase is also a key finding of CoCC [3]. Prolonged contrast effects have been linked to stromal components, so tumors with high levels of fibrous stroma are thought to show extended periods of contrast effects [9]. In addition, portal vein and hepatic artery penetration into the tumor play an important role in making a precise diagnosis of CoCC [4, 9]. Curative surgery results in better prognosis than chemotherapy and hepatic arterial infusion; the 5-year survival rate from 28 curative resections of CoCC was approximately 75% compared to only 33% for ICC, which illustrates a promising long-term prognosis [5].

A characteristic histopathological feature of CoCC is the absence of mucus production, which is important in distinguishing CoCCs from ICCs [10]. In this particular case, immunohistochemistry, CK-7, CK-19, EMA, and NCAM1 staining were useful for diagnosis [11, 12]. Among them, EMA and NCAM1 are particularly important [12]. EMA staining in the glandular lumen and a positive NCAM1 result are observed in CoCCs, while EMA staining in the cytoplasm and a negative NCAM1 result are usually observed in ICCs [13]. Thus, this patient was diagnosed with CoCC due to the presence of mild nuclear atypia forming small tubular glands, the absence of mucus production, a positive EMA result, and a positive NCAM1 result.

There were 78 reported cases of “cholangiolocellular carcinoma” in Japan between 2008 and 2020. Five cases required more than 1 year of follow-up from initial diagnosis to surgery (Table1) [14–18]. CoCC has a slow tumor doubling time of 285 days compared to ICC of 70 days [19]. This gradual progression pattern is also characteristic of CoCC in our patient. Our case showed very slow progression, having a 5-year follow-up period from first detection to surgery. Among the reported cases, five out of six patients were diagnosed with hemangioma upon admission, and one of the six patients was diagnosed with inflammatory pseudo-tumor. The radiological findings of CoCC were similar to both hemangioma and inflammatory pseudo-tumor, requiring long-term follow-up.

**Table 1:** Cholangiolocarcinoma cases followed-up for more than 12 months from first admission to surgery

No	Year	Age	Sex	Etiology	First diagnosis	Observation period (month)	Operative method	First size (mm)	Resected specimen	Prognosis
1	2009	76	M	HCV	n.d	31	Sub-segmentectomy	10 mm	n.d	n.d
2	2012	69	M	HBV	Hemangioma	48	Sub-segmentectomy	12 mm	41 × 25 mm	n.d
3	2015	41	F	HBV	Hemangioma	24	Extended right lobectomy	18 × 17 mm	19 × 15 × 12 mm	Alive (30 M)
4	2015	74	F	NASH	Inflammatory pseudo-tumor	32	Extended right lobectomy	38 mm	68 mm	Alive (32 M)
5	2018	73	F	HCV	Hemangioma	16	S3 sub-segmentectomy	10 mm	65 × 55 × 34 mm	Alive (20 M)
6	Our case	66	M	NASH	Hemangioma	60	Left lobectomy	10 × 5 mm	15 × 15 × 15 mm	Alive (12 M)

*HCV* hepatitis C virus, *HBV* hepatitis B virus, *NASH* non-alcoholic steatohepatitis, *n.d* not detected

In conclusion, it is difficult to distinguish CoCC from other benign tumors, such as hemangiomas and inflammatory pseudo-tumors during initial staging because of the similarity of their radiological findings. As such, it is necessary not to overlook CoCC during the follow-up period.

## Data Availability

Data sharing is not applicable for this article as no data sets were generated nor analyzed for the study.

## Abbreviations

CEA: Carcinoembryonic antigen
CK: Cytokeratin
CoCC: Cholangiolocellular carcinoma
CT: Computed tomography
EMA: Epithelial membrane antigen
HBV: Hepatitis B virus
HCV: Hepatitis C virus
HPCs: Hepatic progenitor cells
ICC: Intrahepatic cholangiocarcinoma
NASH: Non-alcoholic steatohepatitis
NCAM1: Neural cell adhesion molecule 1
WHO: World Health Organization
